# Cryptogenic Stroke Caused by a Newly Diagnosed Patent Foramen Ovale in a Healthy Young Adult

**DOI:** 10.7759/cureus.46895

**Published:** 2023-10-12

**Authors:** Esosa U Ukponmwan, Sandeep Banga, Andrew G Kim, Mohammed Qintar, George Abela

**Affiliations:** 1 Internal Medicine, Michigan State University, East Lansing, USA; 2 Cardiology, Michigan State University, East Lansing, USA; 3 Cardiology, Human Medicine, Michigan State University, East Lansing, USA

**Keywords:** magnetic resonance imaging, meningioma, embolism and thrombosis, paradoxical embolism, transcatheter repair, transcatheter, amplatzer septal occluder, cryptogenic strokes, patent foramen ovale (pfo)

## Abstract

The foramen ovale serves as an opening between the right and left atria at the site of the fossa ovalis in the fetus during uterine life. During fetal life, it makes it possible for venous blood from the maternal placenta with oxygen and nutrients to bypass the immature fetal lung and get transported to the left side of the heart and onto the systemic circulation. This hole from the right to the left atrium is usually occluded at the time of birth or shortly after birth, due to increased pressures in the left-sided cardiac cavities associated with normal breathing during delivery or shortly afterwards. If the foramen ovale remains open and fails to fuse beyond the first year of life, it is known as a patent foramen ovale (PFO). PFO occurs when, during fetal life, the septum primum and secundum, which develop and overlap normally, fail to fuse at birth. This results in the persistence of communication between the right and left atria. Paradoxical embolism from the right to the left side of the heart can occur through a PFO, causing a cryptogenic stroke or embolic stroke of an undetermined source in an otherwise healthy adult. There was a debate on the long-term benefits of closure. However, data from the randomized evaluation of the recurrent stroke comparing PFO closure to established current standard of care treatment (RESPECT) trial and two randomized trials (patent foramen ovale closure or anticoagulants versus antiplatelet therapy to prevent stroke recurrence (CLOSE) and reduction by dutasteride of prostate cancer events (REDUCE)) have clarified that there is a benefit to closure. In this case report, we describe a patient who presented with cryptogenic stroke, the investigations, imaging modalities for diagnosis of PFO, and procedure for closure. We also describe long-term outcomes and management following closure.

## Introduction

The foramen ovale serves as a small opening from the right to the left atria located at the region of the fossa ovalis within the interatrial septum during uterine life. It functions during fetal life to enable venous blood from the maternal placenta with oxygen and nutrients to bypass the immature fetal lung and travel directly to the left side of the heart, to be pumped to the rest of the systemic circulation [1,2]. The communication usually closes after birth because of the increased pressure in the left-sided cardiac cavities associated with normal breathing at the time of delivery. If the foramen ovale persists beyond the age of one, it is called a patent foramen ovale (PFO) [1]. PFO occurs when during fetal development the septum primum and secundum, which develop and overlap normally, fail to fuse at birth [2].

PFO is a hemodynamically insignificant interatrial communication, which is estimated from imaging with transthoracic and transesophageal echocardiogram, transcranial Doppler, and autopsy samples to be present in greater than 25% of the adult population [1-3]. In 75% of individuals, it closes shortly after birth; however, it may persist in 20%-34% of the adult population and be a conduit for paradoxical emboli leading to stroke [1-3]. We present and discuss the management of a patient with cryptogenic stroke who presented to our center.

## Case presentation

A 45-year-old healthy male, with no significant past medical history, other than an incidentally diagnosed methyl tetrahydrofolate reductase deficiency, presented to our facility via the emergency services with a chief complaint of sudden onset of blurred vision, dizziness, and left arm and leg weakness with associated nausea and vomiting, which started on the morning of presentation.

A review of systems was positive for fatigue, blurred and double vision, falls, and headaches. His physical examination was notable for left homonymous superior quadrantanopia with loss of left upper eye field, and he also had a left finger tapping incoordination, but an otherwise normal physical examination. He had a mild elevation in his blood pressure and tachycardia; otherwise, vital signs were normal. The electrocardiogram showed a normal sinus rhythm, and laboratory investigations were all within normal limits. Bilateral lower extremity ultrasound for deep vein thrombosis was negative.

**Figure 1:**
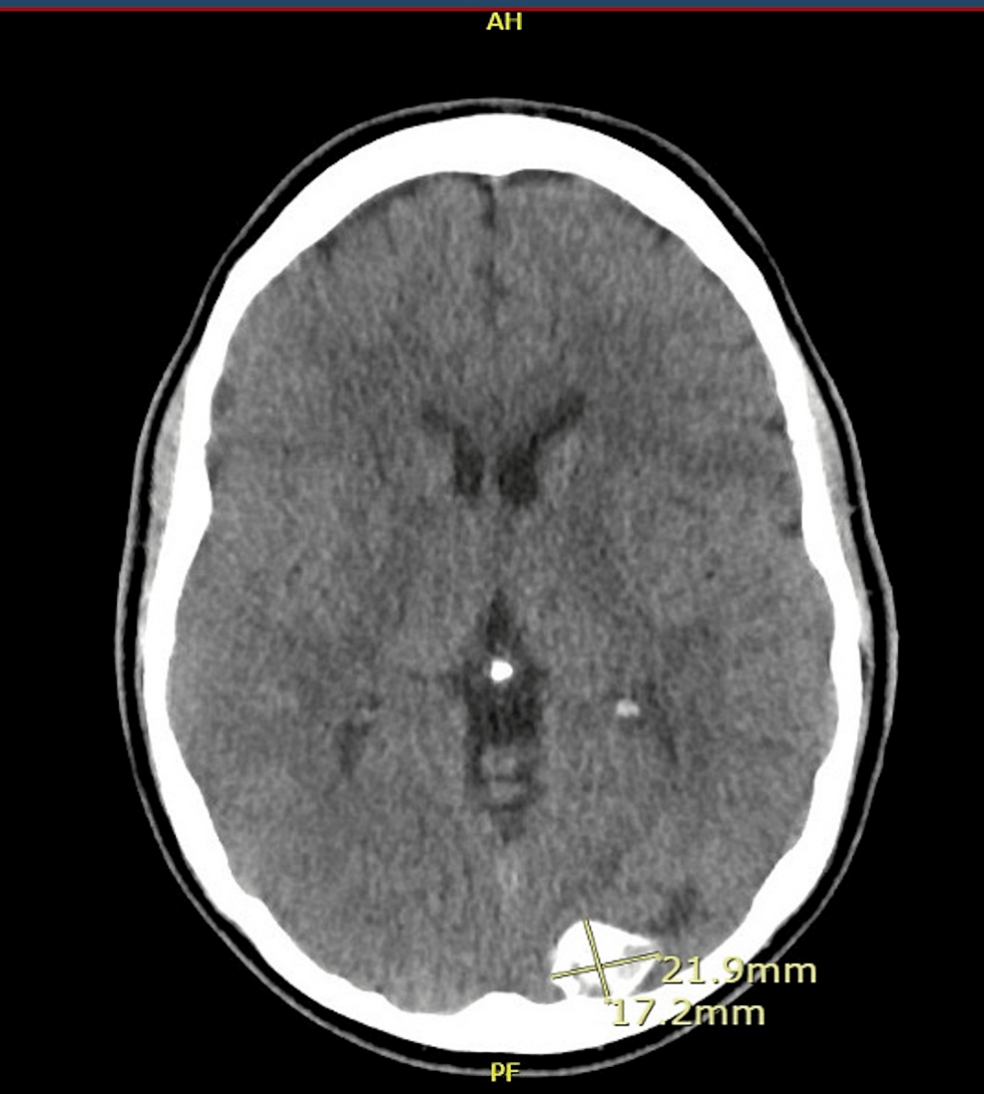
CT scan of the brain showing a meningioma

A CT scan of the brain at presentation showed a 21.9 x 28.2 x 17.2 mm left occipital mass, suggestive of a meningioma; otherwise, no other abnormality was noted. This was thought to be a stable lesion by the neurosurgery team, not related to the present presentation. An image of the CT scan of the brain at presentation is shown in Figure 1.

**Figure 2:**
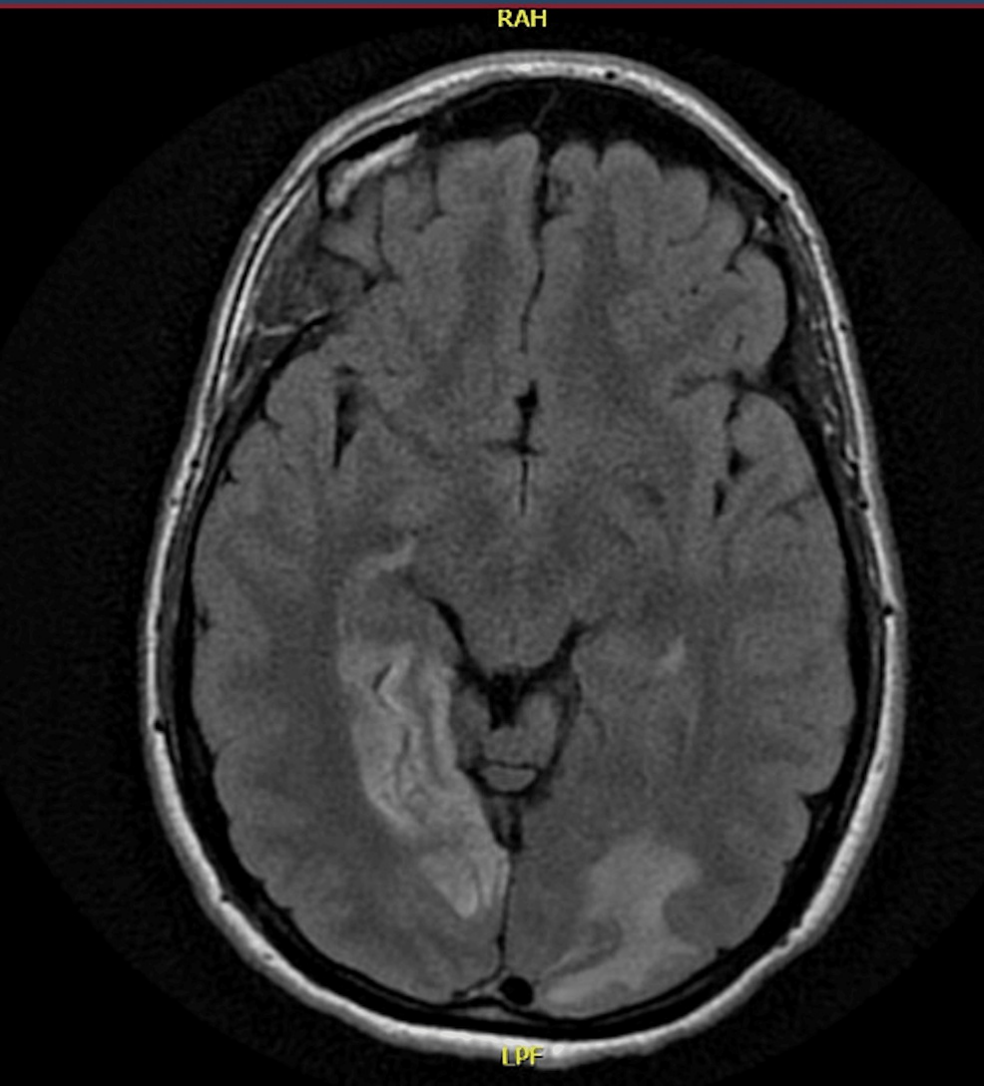
MRI done showing acute-subacute, mildly enhancing ischemia/infarction along the posterior medial right temporal and occipital lobe cortices

Following the CT scan of the brain, a magnetic resonance imaging (MRI) of the brain was done, which showed acute ischemia of the posterior medial right temporal and occipital cortices, as shown in the figure below (Figure 2).

**Figure 3:**
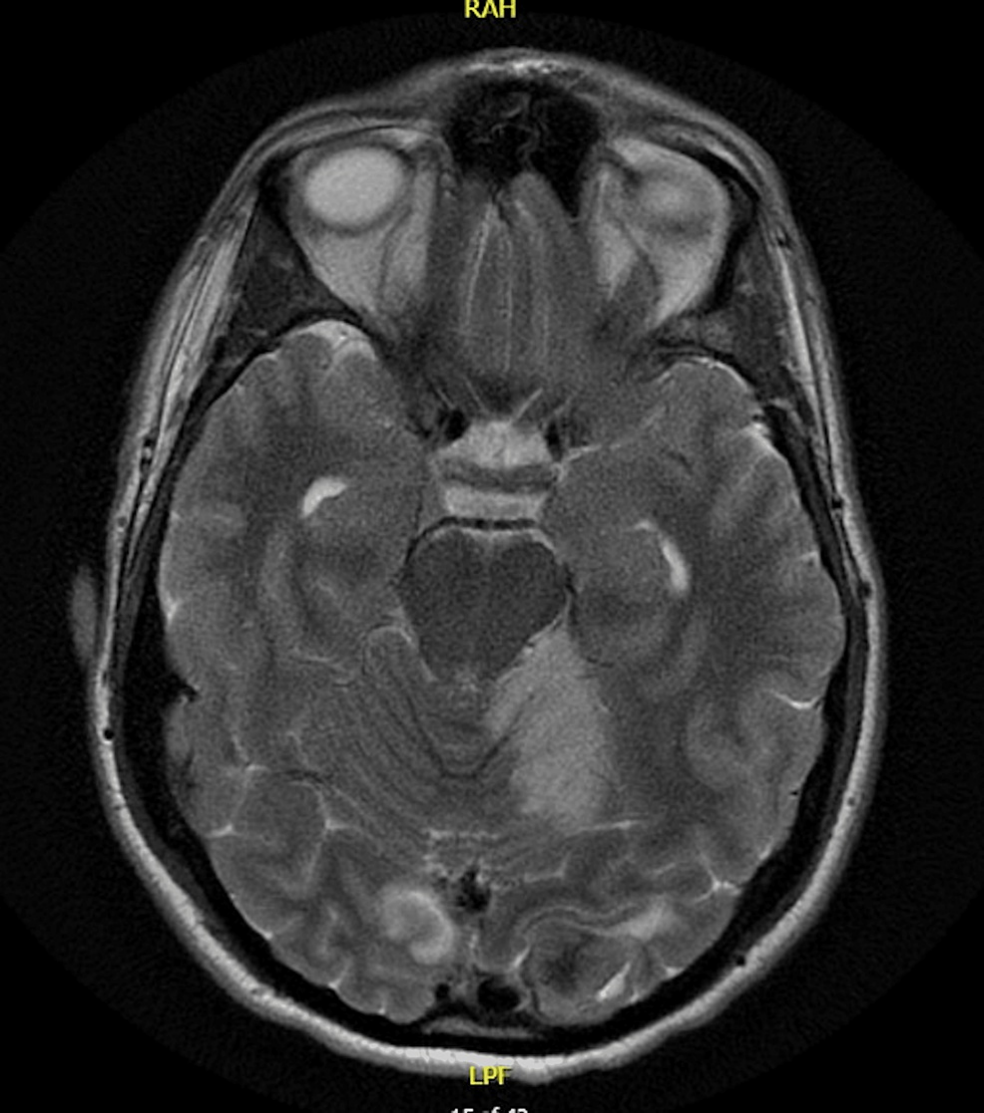
MRI of the brain showing left occipital meningioma, acute ischemia/infarction with mild hemorrhage along the left vermis and cerebellum

MRI also showed acute ischemic infarction with mild hemorrhage along the left vermis and cerebellum. It also demonstrated a meningioma in the left occipital lobe, as shown below (Figure 3).

**Figure 4:**
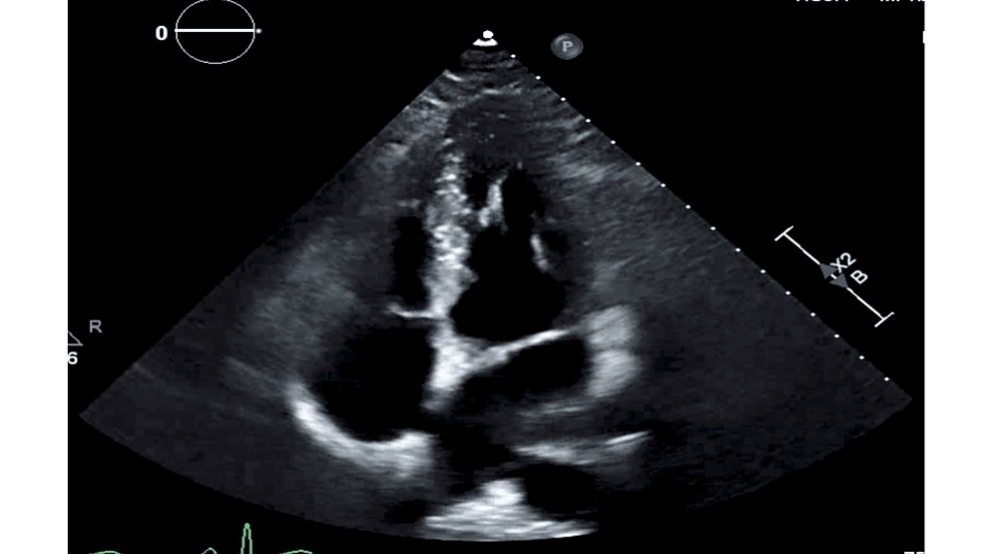
Echocardiogram done at presentation showing the communication between the right and left atria via the patent foramen ovale

Our patient had a normal left ventricular ejection fraction of 60%-65% and normal cardiac valves on transthoracic echocardiogram. Transthoracic and transesophageal echocardiogram demonstrated an aneurysmal inter-atrial septum with inter-atrial communication on color flow Doppler, consistent with a PFO. The echocardiogram done at presentation is shown with inter-atrial communication via the PFO (Figure 4).

**Figure 5:**
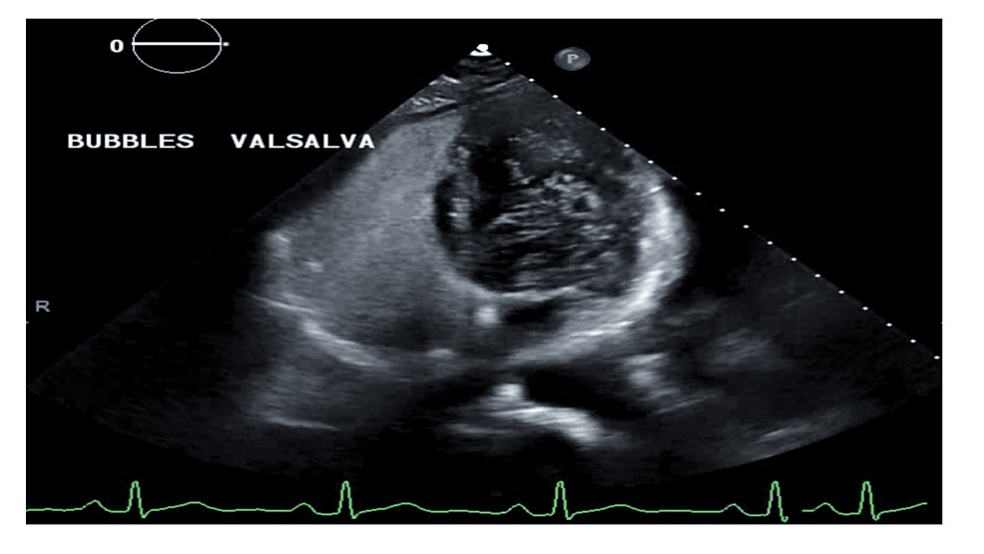
Echocardiogram done showing a positive bubble study on Valsalva suggestive of a patent foramen ovale

Transthoracic echocardiography done had a positive bubble study with a significant number of bubbles crossing to the left heart chamber with Valsalva maneuver, consistent with a PFO. The transthoracic echocardiogram done with a positive bubble study is shown in Figure 5.

A diagnosis of cryptogenic paradoxical cardioembolic stroke due to PFO was made. He was medically stabilized and started on aspirin and atorvastatin at discharge. He was referred to the structural cardiologist for definitive closure of PFO.

The patient followed up with the structural cardiologist and had a repair of the PFO four months later. A transcatheter closure of the PFO was done. Closure of the interatrial communication via the right femoral venous percutaneous approach using a 30 mm Amplatzer Talisman occluder device was done. An intracardiac echocardiography using an 8-French AcuNav ultrasound catheter prior to insertion of the Amplatzer Talisman occluder device demonstrated good anatomy, with normal cardiac valves and normal right and left ventricular function. After ensuring adequate anatomy, the Amplatzer Talisman occluder device was delivered over an Amplatzer wire and introducer, with good capture afterward.

**Figure 6:**
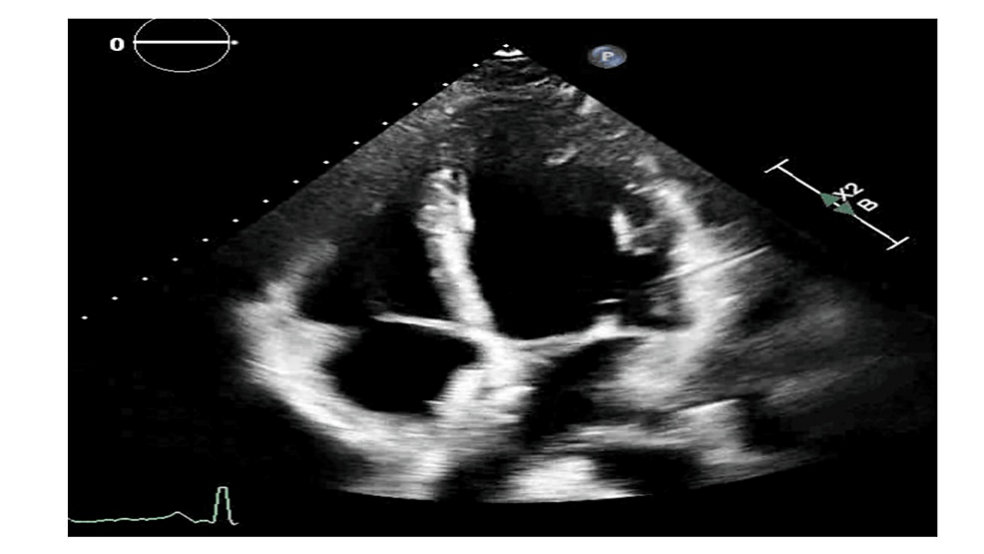
Echocardiogram after PFO closure showing occluder in adequate position traversing the atrial septum without a residual shunt and negative bubble study

Post-delivery intracardiac echocardiogram showed a stable position of the device and no further interatrial communication on color flow Doppler. A post-procedure echocardiogram showed normal ejection fraction and adequate position of the Amplatzer Talisman occluder device with no residual shunt. An image from the post-procedure echocardiogram is shown in Figure 6.

The patient was discharged home on clopidogrel, aspirin, and post-procedure antibiotic prophylaxis. The patient did not develop any complications from the procedure. He did very well afterward and followed up in the clinic with no new strokes, clinical signs, or symptoms.

## Discussion

Several research studies have indicated that PFO occurs in more than 25% of adults [1-3]. This is evidenced by autopsy samples, contrast-enhanced transthoracic and transesophageal echocardiogram, contrast-enhanced transcranial Doppler, and other cardiac imaging studies [1,2].

PFO is usually asymptomatic, as it is distinct from atrial septal defect where there is a hole in either septum that causes communication between both the right and left atria [1,3]. The PFO may open up at times of increased right atrial pressure, such as during the Valsalva maneuver [1,3].

Transthoracic (TT) echocardiography and transesophageal (TE) echocardiography with saline contrast injection are widely used to detect PFO. A PFO is judged to be present if any microbubble is seen in the left-sided cardiac chambers within three cardiac cycles from the maximum right atrial opacification [3].

Our case demonstrates the importance of a thorough workup and describes the management of a young patient with no known prior predisposition to a cerebrovascular accident who presented with cryptogenic stroke. Several studies and metanalysis have demonstrated long-term benefits in the closure of PFO in patients who have indications for closure [4,5]. Some of the indications for closure are a history of cryptogenic stroke, systemic embolization, decompression illness, platypnoea-orthodeoxia syndrome, and migraine with aura [4].

Our patient had a cryptogenic stroke, which is one which, despite extensive investigations, a clear cause cannot be found [3]. A bilateral lower extremity ultrasound yielded a negative result, indicating the absence of deep vein thrombosis (DVT) in the lower limbs and thus ruling out this condition. The presumed cause in a patient with PFO after ruling out other causes such as atherosclerotic disease and carotid dissection is thought to be a paradoxical embolus through the PFO. The mechanism is thought to be due to the translocation of a venous thrombus to the arterial circulation under hemodynamic conditions, where the PFO opens during rapid rise and fall in right atrial pressure such as straining, coughing, and Valsalva maneuver [3].

Paradoxical embolism from the right to the left side of the heart can occur through a PFO, causing a cryptogenic stroke or embolic stroke of an undetermined source in an otherwise healthy adult. There was a debate on the long-term benefits of closure; however, data from the randomized evaluation of the recurrent stroke comparing PFO closure to established current standard of care treatment (RESPECT) trial and two randomized trials (patent foramen ovale closure or anticoagulants versus antiplatelet therapy to prevent stroke recurrence (CLOSE) and reduction by dutasteride of prostate cancer events (REDUCE)) and several meta-analyses of these studies have clarified that there is a benefit to the closure of PFO [5-9].

The result of the RESPECT trial, which used the Amplatzer PFO Occluder with the transcatheter approach, showed long-term (at 5.9 years) significant reduction in cardiovascular end-point of death, stroke, or transient ischemic attack compared to medical therapy alone (HR 0.55; 95% CI (0.31-0.99; p=0.046) [4-8].

A number of randomized trials have demonstrated that PFO closure is superior to medical therapy with antiplatelet therapy alone [9-11]. Multiple studies, including a meta-analysis of multiple trials (CLOSE 2017, CLOSURE 2013, RESPECT 2013, and PC 2013), showed that, in patients who had a cryptogenic stroke, thought to be due to a PFO, those assigned to device closure and antiplatelet therapy had reduced incidence of recurrent strokes and brain infarctions compared to those assigned to antiplatelet therapy alone [9-11]. In one study by Sondergaard et al., device-related complications occurred in 1.6% of the PFO closure group and 6.6% developed atrial fibrillation [10]. In another study by Mas et al., complications related to the device occurred in 4.6% of the device closure group, and transient atrial fibrillation occurred in 5.9% of the device closure group [11]. Atrial fibrillation is a reported complication after closure in multiple studies [9-11]. However, the benefits of device closure to prevent recurrent strokes outweighed the risk of complications [9-11]. Findings from these studies align with findings from the RESPECT and CLOSE trials.

Patients who present with complications of PFO, such as embolic stroke, should be assessed for closure to prevent or reduce the chances of a subsequent stroke. Assessment for closure is usually based on the risk of pulmonary embolism (RoPE) score in patients with cryptogenic stroke [2,12]. The RoPE score applies Bayes' theorem and calculates the probability that PFO is causally related to stroke (PFO attributable fraction (PFOAF)), with higher scores implying a greater possibility that a PFO is etiologically associated with a cryptogenic stroke [2]. Factors included in the RoPE score are diabetes, hypertension, smoking, and prior stroke or transient ischemic attack (TIA.) The 10-point risk of paradoxical embolism score is calculated from these variables so that the youngest patients with superficial strokes and without vascular risk factors have the highest scores [2,11]. Our patient had a RoPE score of 8 points, which corresponds to an 84% chance of stroke due to a PFO.

Our patient had a PFO closure after the incident stroke. He was continued on antiplatelet therapy and has followed up afterward, with no repeat stroke episodes.

## Conclusions

Patent foramen management with device occlusion is superior to medical therapy alone with anti-platelet medications for long-term management of symptomatic PFO to prevent recurrent strokes or brain infarcts. Evaluation and subsequent closure should be included as part of the routine management of patients who present with cryptogenic paradoxical or systemic embolism, thought to be due to the presence of a PFO.

More studies need to be done in asymptomatic patients with PFO, who have no clear indication for closure such as a history of cryptogenic stroke, systemic embolization, decompression illness, platypnoea-orthodeoxia syndrome, and migraine with aura, to assess the long-term benefits of closure versus medical therapy alone to prevent strokes in the future.
